# Supplementary material for: Iterative improvement in the automatic modular design of robot swarms
Source: PeerJ Comput Sci. 2020 Dec 7;6:e322. doi: 10.7717/peerj-cs.322 (PMC7924708; doi:10.7717/peerj-cs.322)
Supplement: Supplemental Information 3 [file peerj-cs-06-322-s003.zip › argos3/doc/api/standalone/a00552_source.html]

ARGoS: plugins/robots/mini-quadrotor/simulator/miniquadrotor\_rotor\_default\_actuator.cpp Source File


- Main Page
- Related Pages
- Namespaces
- Classes
- Files

- File List
- File Members

# plugins/robots/mini-quadrotor/simulator/miniquadrotor\_rotor\_default\_actuator.cpp

Go to the documentation of this file.

```
00001 
00007 #include "miniquadrotor_rotor_default_actuator.h"
00008 #include <argos3/plugins/simulator/entities/rotor_equipped_entity.h>
00009 #include <argos3/core/utility/logging/argos_log.h>
00010 #include <argos3/core/utility/plugins/factory.h>
00011 
00012 namespace argos {
00013 
00014    /****************************************/
00015    /****************************************/
00016 
00017    CMiniQuadrotorRotorDefaultActuator::CMiniQuadrotorRotorDefaultActuator() :
00018       m_pcRotorEquippedEntity(NULL),
00019       m_pcRNG(NULL),
00020       m_fNoiseStdDeviation(0.0f) {
00021    }
00022    
00023    /****************************************/
00024    /****************************************/
00025    
00026    void CMiniQuadrotorRotorDefaultActuator::SetRobot(CComposableEntity& c_entity) {
00027       try {
00028          m_pcRotorEquippedEntity = &(c_entity.GetComponent<CRotorEquippedEntity>("rotors"));
00029          if(m_pcRotorEquippedEntity->GetNumRotors() != 4) {
00030             THROW_ARGOSEXCEPTION("The mini-quadrotor rotor actuator can be associated only to a mini-quadrotor");
00031          }
00032       }
00033       catch(CARGoSException& ex) {
00034          THROW_ARGOSEXCEPTION_NESTED("Error setting mini-quadrotor rotor actuator to entity \"" << c_entity.GetId() << "\"", ex);
00035       }
00036    }
00037 
00038    /****************************************/
00039    /****************************************/
00040 
00041    void CMiniQuadrotorRotorDefaultActuator::Init(TConfigurationNode& t_tree) {
00042       try {
00043          CCI_MiniQuadrotorRotorActuator::Init(t_tree);
00044          GetNodeAttributeOrDefault<Real>(t_tree, "noise_std_dev", m_fNoiseStdDeviation, 0.0f);
00045          if(m_fNoiseStdDeviation > 0.0f) {
00046             m_pcRNG = CRandom::CreateRNG("argos");
00047          }
00048       }
00049       catch(CARGoSException& ex) {
00050          THROW_ARGOSEXCEPTION_NESTED("Initialization error in mini-quadrotor rotor actuator.", ex);
00051       }
00052    }
00053 
00054    /****************************************/
00055    /****************************************/
00056    
00057    void CMiniQuadrotorRotorDefaultActuator::SetRotorVelocities(const CCI_MiniQuadrotorRotorActuator::SVelocities& s_velocities) {
00058       /* Set velocities */
00059       m_sCurrentVelocities = s_velocities;
00060       /* Apply noise */
00061       if(m_fNoiseStdDeviation > 0.0f) {
00062          AddGaussianNoise();
00063       }
00064    }
00065    
00066    /****************************************/
00067    /****************************************/
00068    
00069    void CMiniQuadrotorRotorDefaultActuator::Update() {
00070       m_pcRotorEquippedEntity->SetVelocities(m_sCurrentVelocities.Velocities);
00071    }
00072 
00073    /****************************************/
00074    /****************************************/
00075    
00076    void CMiniQuadrotorRotorDefaultActuator::Reset() {
00077       m_sCurrentVelocities = SVelocities();
00078    }
00079    
00080    /****************************************/
00081    /****************************************/
00082    
00083    void CMiniQuadrotorRotorDefaultActuator::AddGaussianNoise() {
00084       m_sCurrentVelocities.Velocities[0] += m_sCurrentVelocities.Velocities[0] * m_pcRNG->Gaussian(m_fNoiseStdDeviation);
00085       m_sCurrentVelocities.Velocities[1] += m_sCurrentVelocities.Velocities[1] * m_pcRNG->Gaussian(m_fNoiseStdDeviation);
00086       m_sCurrentVelocities.Velocities[2] += m_sCurrentVelocities.Velocities[2] * m_pcRNG->Gaussian(m_fNoiseStdDeviation);
00087       m_sCurrentVelocities.Velocities[3] += m_sCurrentVelocities.Velocities[3] * m_pcRNG->Gaussian(m_fNoiseStdDeviation);
00088    }
00089 
00090    /****************************************/
00091    /****************************************/
00092 
00093 }
00094 
00095 REGISTER_ACTUATOR(CMiniQuadrotorRotorDefaultActuator,
00096                   "miniquadrotor_rotor", "default",
00097                   "Carlo Pinciroli [ilpincy@gmail.com]",
00098                   "1.0",
00099                   "The mini-quadrotor rotor actuator.",
00100                   "This actuator controls the four rotors of a mini-quadrotor robot. For a\n"
00101                   "complete description of its usage, refer to the\n"
00102                   "ci_miniquadrotor_rotor_actuator.h file.\n\n"
00103                   "REQUIRED XML CONFIGURATION\n\n"
00104                   "  <controllers>\n"
00105                   "    ...\n"
00106                   "    <my_controller ...>\n"
00107                   "      ...\n"
00108                   "      <actuators>\n"
00109                   "        ...\n"
00110                   "        <miniquadrotor_rotor implementation=\"default\" />\n"
00111                   "        ...\n"
00112                   "      </actuators>\n"
00113                   "      ...\n"
00114                   "    </my_controller>\n"
00115                   "    ...\n"
00116                   "  </controllers>\n\n"
00117                   "OPTIONAL XML CONFIGURATION\n\n"
00118                   "It is possible to specify noisy speed in order to match the characteristics\n"
00119                   "of the real robot. This can be done with the attribute: 'noise_std_dev',\n" 
00120                   "which indicates the standard deviation of a gaussian noise applied to the\n"
00121                   "desired velocity of the rotor:\n\n"
00122                   "  <controllers>\n"
00123                   "    ...\n"
00124                   "    <my_controller ...>\n"
00125                   "      ...\n"
00126                   "      <actuators>\n"
00127                   "        ...\n"
00128                   "        <miniquadrotor_rotor implementation=\"default\"\n"
00129                   "                               noise_std_dev=\"1\" />\n"
00130                   "        ...\n"
00131                   "      </actuators>\n"
00132                   "      ...\n"
00133                   "    </my_controller>\n"
00134                   "    ...\n"
00135                   "  </controllers>\n",
00136                   "Usable"
00137    );
00138
```

---

Generated on 10 Jul 2018 for ARGoS by 
 1.6.1
